# Centromere localization and function of Mis18 requires Yippee‐like domain‐mediated oligomerization

**DOI:** 10.15252/embr.201541520

**Published:** 2016-03-03

**Authors:** Lakxmi Subramanian, Bethan Medina‐Pritchard, Rachael Barton, Frances Spiller, Raghavendran Kulasegaran‐Shylini, Guoda Radaviciute, Robin C Allshire, A Arockia Jeyaprakash

**Affiliations:** ^1^ Wellcome Trust Centre for Cell BiologyInstitute of Cell BiologyUniversity of EdinburghEdinburghUK

**Keywords:** CENP‐A, centromere, epigenetics, Mis18, Yippee, Cell Cycle, DNA Replication, Repair & Recombination, Structural Biology

## Abstract

Mis18 is a key regulator responsible for the centromere localization of the CENP‐A chaperone Scm3 in *Schizosaccharomyces pombe* and HJURP in humans, which establishes CENP‐A chromatin that defines centromeres. The molecular and structural determinants of Mis18 centromere targeting remain elusive. Here, by combining structural, biochemical, and yeast genetic studies, we show that the oligomerization of *S. pombe* Mis18, mediated via its conserved N‐terminal Yippee‐like domain, is crucial for its centromere localization and function. The crystal structure of the N‐terminal Yippee‐like domain reveals a fold containing a cradle‐shaped pocket that is implicated in protein/nucleic acid binding, which we show is required for Mis18 function. While the N‐terminal Yippee‐like domain forms a homodimer *in vitro* and *in vivo*, full‐length Mis18, including the C‐terminal α‐helical domain, forms a homotetramer *in vitro*. We also show that the Yippee‐like domains of human Mis18α/Mis18β interact to form a heterodimer, implying a conserved structural theme for Mis18 regulation.

## Introduction

The accurate distribution of genetic information to daughter cells during cell division relies on the physical attachment of chromosomes to spindle microtubules mediated by kinetochores. Kinetochores are large protein assemblies deposited at specific chromosomal loci known as centromeres 1, 2, 3. Defective centromere function results in chromosome segregation errors that can contribute to genomic instability implicated in cancer 4. Hence, understanding the molecular mechanisms that promote kinetochore establishment and maintenance at centromeres is of prime importance.

The location of most eukaryotic centromeres is determined by the assembly of specialized chromatin composed of nucleosomes in which canonical histone H3 is replaced by the centromere‐specific H3 variant CENP‐A in vertebrates and Cnp1 (CENP‐A^Cnp1^) in *Schizosaccharomyces pombe* 3, 5. Thus, the establishment and maintenance of kinetochores requires CENP‐A to be recruited to and deposited at centromeres. In *S. pombe*, CENP‐A^Cnp1^ is specifically incorporated into chromatin over the central domain of endogenous centromeres where it is flanked by heterochromatin formed on outer repeat elements 6, 7.

During S phase, CENP‐A^Cnp1^ levels at fission yeast centromeres are halved following DNA replication. Subsequently, CENP‐A^Cnp1^ levels are replenished during G2 phase of the cell cycle 8. In early mitosis, the Mis18 complex comprising Mis18, Mis16, Eic1/Mis19/Kis1, and Eic2/Mis20, along with the CENP‐A^Cnp1^ chaperone Scm3 (counterpart of vertebrate HJURP; HJURP^Scm3^), dissociates from centromeres and re‐associates in mid/late anaphase following chromosome segregation 9, 10, 11, 12, 13, 14, 15. Exclusion of the Mis18 complex and HJURP^Scm3^ from centromeres during mitosis likely provides an opportunity for the CENP‐A^Cnp1^ loading cycle to reset and thereby prevent continual CENP‐A^Cnp1^ deposition 16. HJURP^Scm3^ directly interacts with CENP‐A^Cnp1^ and is essential for the deposition of new CENP‐A^Cnp1^ at centromeres 14. Genetic studies have shown that Mis16, Mis18, and Eic1/Mis19/Kis1 are essential genes that are required for the localization of HJURP^Scm3^ to centromeres and hence CENP‐A^Cnp1^ maintenance at centromeres 10, 11, 13, 14, 17.

Most of the components of the CENP‐A assembly pathway are conserved among eukaryotes, but with a few striking differences. Humans possess two isoforms of Mis18 (Mis18α and Mis18β) and Mis16 (RbAp46 and RbAp48) 10. During telophase, the human Mis18 complex comprising Mis18α, Mis18β, and Mis18BP1/KNL2, along with RbAp46 and RbAp48, associates with centromeres 17. Mis18BP1/KNL2 has no detectable *S. pombe* homolog, but Eic1/Mis19/Kis1 appears to perform an analogous function 11, 13. As in *S. pombe*, the human Mis18 complex is required for HJURP recruitment to centromeres, where CENP‐A is deposited during early G1 rather than G2 9. In addition, the stable incorporation of CENP‐A at human centromeres requires the small GTPase activity of Cdc42 regulated by MgcRacGap/ECT2 18.

Although key conserved players involved in the assembly and maintenance of CENP‐A chromatin at centromeres have been identified, the molecular mechanisms through which they exert their function remain unclear. Mis18 is critical for the specification of centromeres from fission yeast to humans, however, what allows Mis18 to regulate centromere specification remains largely unknown. To gain insights into the structural features of *S. pombe* Mis18 that allow it to bind centromeres and promote HJURP^Scm3^ recruitment and CENP‐A^Cnp1^ assembly, we determined the crystal structure of its highly conserved “Yippee‐like” N‐terminal globular domain. Our structural and biochemical analyses reveal that the Mis18 “Yippee‐like” domain possesses a fold that is implicated in protein/nucleic acid binding and that this domain has an innate tendency to homodimerize both *in vitro* and *in vivo*. However, full‐length Mis18 forms a homotetramer *in vitro,* highlighting a role for the C‐terminal α‐helical domain in influencing the overall oligomeric state of the protein. Genetic analyses using structure‐guided mutants demonstrate that dimerization of the “Yippee‐like” domain is essential for the centromere localization and hence the function of Mis18.

## Results and Discussion

### *sp*Mis18 possesses an N‐terminal Yippee‐like globular domain that is implicated in protein/nucleic acid binding

**Figure 1:**
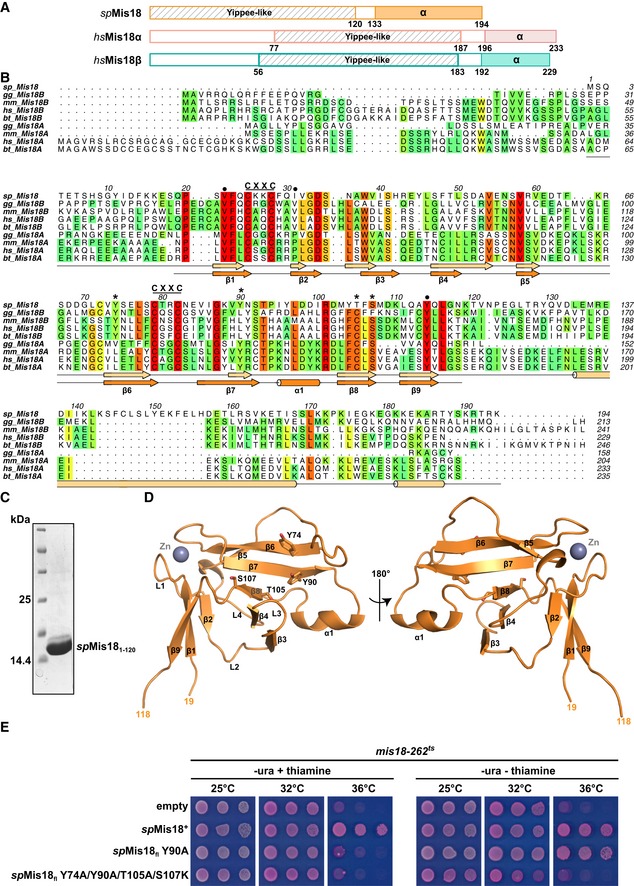
*sp*Mis18 possesses a highly conserved N‐terminal Yippee‐like globular domain and a C‐terminal α‐helical domain Schematic representation of domain organization of Mis18 proteins from *Schizosaccharomyces pombe* and human (as suggested by the Conserved Domain Database (CDD) and secondary structure predictions).Amino acid conservation of Mis18 proteins among eukaryotes. The alignment includes orthologs from *S. pombe* (*sp*), *G. gallus* (*gg*), *M. musculus* (*mm*), *H. sapiens* (*hs*), and *B. taurus* (*bt*). Predicted (light orange; using PsiPred, http://bioinf.cs.ucl.ac.uk/psipred) and observed (bright orange; from the crystal structure shown in D) secondary structure elements are shown below the aligned sequences. Amino acid residues mutated in this study are highlighted with circles (dimer interface residues) and asterisks (putative substrate‐binding pocket residues).
SDS–PAGE showing a representative fraction of the purified *sp*Mis18 N‐terminal Yippee‐like globular domain (amino acid residues 1–120; *sp*Mis18_1–120_).Cartoon representation of the crystal structure of *sp*Mis18_1–120_ in two different orientations, highlighting key residues within the putative substrate‐binding pocket.Substrate‐binding pocket mutations Y90A and Y74A/Y90A/T105A/S107K affect the ability of ectopically expressed *sp*Mis18_fl_ to rescue the temperature sensitivity of *mis18‐262* cells to varying degrees. Fivefold serial dilutions of *mis18‐262* cells transformed with plasmids harboring the indicated *sp*Mis18_fl_ constructs, spotted on PMG − uracil + phloxine B media supplemented with (repressed) or without (expressed) thiamine, and incubated at the indicated temperatures; dead cells stain dark pink.

**Figure EV1:**
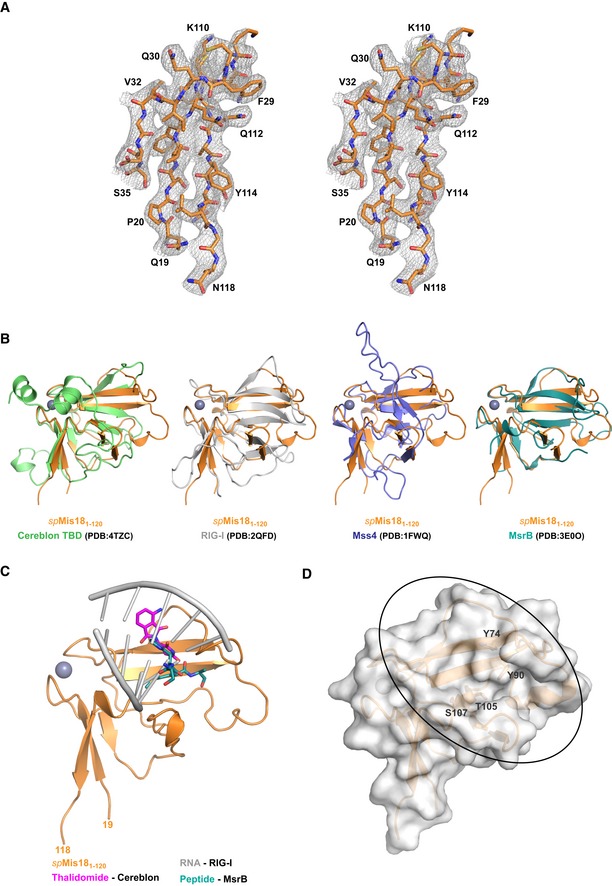
Structural characterization of the N‐terminal Yippee‐like globular domain of *sp*Mis18 Stereo image of electron density map (2*F*_o_–*F*_c_, contoured at 1σ) corresponding to β‐sheet I.Structural superposition of *sp*Mis18_1–120_ onto its closest structural homologs, Cereblon TBD (PDB: 4TZC), RIG‐I (PDB: 2QFD), Mss4(PDD: 1FWQ), and MsrB (PDB: 3E0O). Structural superpositions were carried out using PDBefold web server (http://www.ebi.ac.uk/msd-srv/ssm).Structural superposition of *sp*Mis18_1–120_ with its structural homologs bound to their substrates. For clarity, structural homologs of *sp*Mis18_1–120_ are not shown.Surface representation of *sp*Mis18_1–120_ where the cradle‐shaped pocket and amino acid residues mutated in the complementation assay (Fig 1E) are highlighted.

Amino acid sequence and the predicted secondary structure analysis of *Schizosaccharomyces pombe* Mis18 suggested the presence of a highly conserved N‐terminal globular domain (residues 1–120; *sp*Mis18_1–120_) mainly comprised of β‐strands, followed by a moderately conserved C‐terminal α‐helical domain (residues 121–end; *sp*Mis18_C‐term‐α_) (Fig 1A and B). Mis18_1–120_ shares about 20% sequence similarity with a putative Zn^2+^‐binding protein of unknown function named Yippee, originally identified in *Drosophila*, that is well conserved from yeast to humans 19. *sp*Mis18_1–120_ has two conserved C‐X‐X‐C motifs, which are signature motifs present in metal ion‐binding proteins. Previous analysis showed that mutations within the C‐X‐X‐C motifs of human Mis18α perturb its centromere localization, highlighting the essential role of this domain 17. To structurally characterize *sp*Mis18_1–120_, we purified recombinant *sp*Mis18_1–120_ as a mono‐disperse sample and obtained crystals that diffracted X‐rays to about 2.6 Å (Fig 1C). X‐ray fluorescence scans of the crystals revealed the presence of bound Zn^2+^ ions. The structure was determined by single anomalous dispersion (SAD) exploiting the Zn anomalous signal from the bound Zn^2+^ ions in the crystals. The refined structure has an R factor of 22 and R‐free factor of 26 with good stereochemistry (Table 1 and Fig EV1A). The final model includes amino acid residues 19–118 of *sp*Mis18_1–120_ (Fig 1D). The N‐terminal 18 and C‐terminal 2 (119 and 120) residues are presumably disordered and hence could not be modeled.

**Table 1:** Data collection, phasing, and refinement statistics

	Dataset I	Dataset II
Data collection		
Space group	P3_1_12	P3_1_12
Cell dimensions		
* a*,* b*,* c* (Å)	122.18, 122.18, 73.48	121.07, 121.07, 73.21
α, β, γ (°)	90, 90, 120	90, 90, 120
	*Peak*	
Wavelength	1.2825	0.97625
Resolution (Å)	47.0–3.9 (4.0–3.9)	104.9–2.6 (2.8–2.6)
*R*_merge_	9.5 (98.3)	6.5 (57.3)
*I*/σ*I*	15.3 (3.7)	9.7 (1.8)
Completeness (%)	97.2 (93.9)	99.9 (99.9)
Redundancy	9.9 (10.3)	4.1 (4.1)
Refinement		
Resolution (Å)		39.6–2.6
No. reflections		17,128
*R*_work_/*R*_free_		22.0/26.0
No. atoms		
Protein		2,374
*B*‐factors		
Protein		98.0
R.m.s deviations		
Bond lengths (Å)		0.01
Bond angles (°)		1.75
Ramachandran values		
Favored (%)		93
Disallowed (%)		1

Values in parentheses are for highest‐resolution shell.

The overall fold of *sp*Mis18_1–120_ is formed by antiparallel β‐sheets: a three‐stranded (β1‐β2‐β9: β‐sheet I) and a six‐stranded (β3‐β4‐β8‐β7‐β6‐β5: β‐sheet II) sheet, arranged approximately perpendicular to each other (Fig 1D). The two β‐sheets are held together by a Zn^2+^ ion coordinated via the C‐X‐X‐C motifs from loops L1 and L5 (Fig 1D). Structural comparison of *sp*Mis18_1–120_ with the available structures in the protein data bank (PDB) identified the thalidomide‐binding domain of Cereblon (PDB: 4tzc; Q‐score 20: 0.47; RMSD: 2.12 Å), a component of an E3 ubiquitin ligase complex implicated in DNA repair, replication, and transcription 21, as the closest structural homolog (Fig EV1B). Other proteins that share a similar fold include RIG‐I (PDB: 2qfd; Q‐score: 0.38; RMSD: 2.19 Å), a nucleic acid‐binding protein involved in innate anti‐viral immunity 22; Mss4 (PDB: 1fwq; Q‐score: 0.34; RMSD: 2.03 Å), a guanine nucleotide exchange factor 23; and MsrB (methionine sulfoxide reductase‐B, PDB: 3e0o; Q‐score: 0.32; RMSD: 1.81 Å), an oxidative reductase implicated in aging 24 (Fig EV1B). This is in agreement with a recent bioinformatics study suggesting an evolutionary relationship between Cereblon, Yippee, and Mis18 proteins 25. Although these proteins recognize substrates as diverse as nucleic acids to proteins, they do so via a common cradle‐shaped binding pocket formed by β‐sheet II (Fig EV1C and D). This observation suggested that the putative substrate‐binding site of *sp*Mis18_1–120_ might play an important role in Mis18 function. To test whether the putative substrate‐binding pocket was required for *sp*Mis18 function *in vivo*, we tested the ability of additional *sp*Mis18 expressed from a plasmid to complement the growth phenotype of *mis18‐262* (G117D) cells, which exhibit loss of function for *sp*Mis18 at the restrictive temperature (36°C) 10. While expression of wild‐type *sp*Mis18 restored growth at 36°C, expressing the pocket mutant (Y74A/Y90A/T105A/S107K, Figs 1D and EV1D) failed to complement the loss of *sp*Mis18 function, demonstrating the requirement of this pocket for Mis18 function (Fig 1E).

### *sp*Mis18_1–120_ forms a homodimer

**Figure 2:**
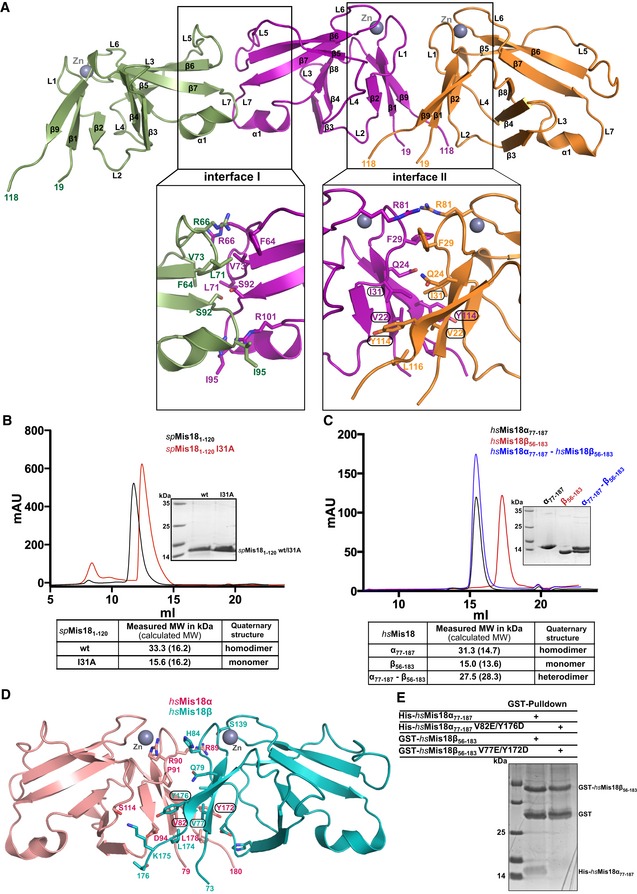
Yippee‐like globular domains of Mis18 proteins possess an intrinsic preference to form homo/heterodimers Cartoon representation of the assembly of *sp*Mis18 Yippee‐like domains (*sp*Mis18_1–120_) as observed in the crystal asymmetric unit. Close‐up views highlight the amino acid composition and interactions stabilizing the dimeric arrangements. Interface I is composed of conserved residues and an extensive binding surface as compared to interface I (see text for details), suggesting dimer II to be a physiologically relevant dimer. Residues mutated in this study are highlighted with circles.Size‐exclusion chromatography (SEC) and SEC‐MALS (SEC combined with multi‐angle light scattering) analyses of the recombinant wild‐type (wt) and dimer‐disrupting mutant (I31A) of *sp*Mis18_1–120_. Measured molecular weights from SEC‐MALS confirm that *sp*Mis18_1–120_ wt is a dimer in solution and *sp*Mis18_1–120_I31A is a monomer.Evaluation of the ability of Yippee‐like domains of human Mis18 proteins (*hs*Mis18α_77–187_ and *hs*Mis18β_56–183_) to form a heterodimer. SEC and SEC‐MALS analyses demonstrate that while *hs*Mis18α_77–187_ elutes at 15.5 ml as a dimer (31.3 kDa), *hs*Mis18β_56–183_ elutes at 17.3 ml as a monomer (15.0 kDa). Purified *hs*Mis18α_77–187_ and *hs*Mis18β_56–183_ when mixed together elute at 15.4 ml as a heterodimer (27.5 kDa).Cartoon representation of the structure of the homology‐modeled human Mis18α_77–187_–Mis18β_56–183_ heterodimer using the crystal structure of *sp*Mis18_1–120_ reported here as a template. Residues mutated in this study are highlighted with circles. Modeling was carried out using Phyre2 web server (www.sbg.bio.ic.ac.uk/phyre2/).
SDS–PAGE analysis of the GST pull‐down assay where wt and dimer‐disrupting mutants of *hs*Mis18α_77–187_ and *hs*Mis18β_56–183_ were co‐expressed as His‐ and GST‐tagged proteins, respectively, in *E. coli*. While wt GST‐*hs*Mis18β_56–183_ showed interaction with wt His‐*hs*Mis18α_77–187_, *hs*Mis18 proteins harboring dimer‐disrupting mutations, GST‐*hs*Mis18β_56–183_V77E/Y172D, and His‐*hs*Mis18α_77–187_V82E/Y176D did not show noticeable interaction. The corresponding Ni‐NTA pull‐downs showing the expression of His‐tagged proteins are shown in Fig EV2D.

**Figure EV2:**
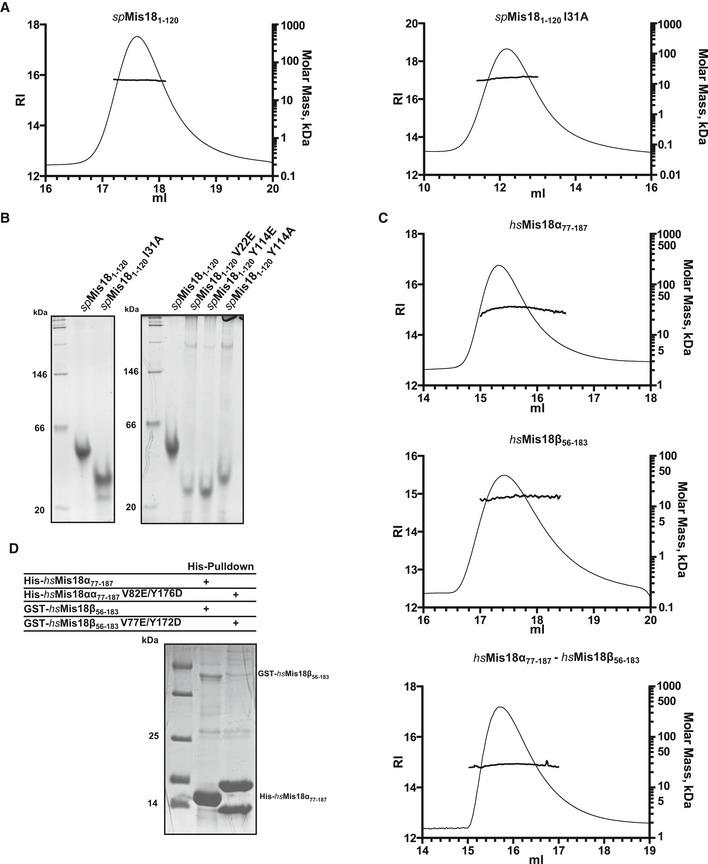
Evaluation of the dimerization ability of Yippee‐like domains of Mis18 proteins SEC‐MALS profiles of His‐*sp*Mis18_1–120_ (left panel) and His‐*sp*Mis18_1–120_I31A (right panel). Refractive index (RI, left *y*‐axis) and molar mass (MW, right *y*‐axis) profiles show that His‐*sp*Mis18_1–120_ (predicted MW of a monomer: 16.2 kDa) is a dimer (measured MW: 33.3 kDa) and His‐*sp*Mis18_1–120_I31A (predicted MW of monomer: 16.2 kDa) is a monomer (measured MW: 15.6 kDa).Native PAGE analysis of dimer interface II mutants. Interface mutants I31A, V22E, Y114E, and Y114A migrated faster than wt *sp*Mis18_1–120_, confirming the effect these mutations have on the oligomeric structure of *sp*Mis18_1–120_.
SEC‐MALS profiles of His‐*hs*Mis18α_77–187_ (top panel), His‐*hs*Mis18β_56–183_ (middle panel), and His‐*hs*Mis18α_77–187_–His‐*hs*Mis18β_56–183_ (bottom panel). While His‐*hs*Mis18α_77–187_ (predicted MW of a monomer: 14.7 kDa) formed a homodimer (measured MW: 31.3 kDa), His‐*hs*Mis18β_56–183_ (predicted MW of a monomer: 13.6 kDa) remained as a monomer (measured MW: 15.0 kDa). His‐*hs*Mis18α_77–187_–His‐*hs*Mis18β_56–183_ (predicted MW of a monomer: 28.3 kDa) formed a heterodimer (measured MW: 27.5 kDa).
SDS–PAGE analysis of Ni‐NTA pull‐down experiment, where His‐*hs*Mis18α_77–187_ and GST‐*hs*Mis18β_56–183_ were co‐expressed in *E. coli* as wt or dimer‐disrupting mutant proteins. While the GST pull‐down assay shown in Fig 2E confirmed the inability of GST‐*hs*Mis18β_56–183_V77E/Y172D to interact with His*‐hs*Mis18α_77–187_V82E/Y176D, the corresponding Ni‐NTA pull‐down shown here confirms the abundant expression of His‐*hs*Mis18α_77–187_V82E/Y176D in the input. We note that His‐*hs*Mis18α_77–187_V82E/Y176D migrates as a doublet. Western blot analysis (data not shown) using anti‐His antibody confirmed the presence of His‐tag on both bands suggesting the unstable nature of His‐*hs*Mis18α_77–187_V82E/Y176D mutant (possibly due to its inability to homo‐ or heterodimerize).

The asymmetric unit of the *sp*Mis18_1–120_ crystals contained three copies of *sp*Mis18_1–120_ assembled in a linear arrangement via two different interfaces (interface I and interface II), resulting in two potential dimeric structures (dimer I and dimer II) (Fig 2A). While the interface stabilizing dimer I is formed by the stacking of loops L5 and L7 of one monomer over their dimeric counterpart, the dimer II interface is formed by the stacking of β‐sheet I (Fig 2A). The oligomeric assembly observed in the crystals prompted us to characterize the oligomeric structure of *sp*Mis18_1–120_ in solution. The molecular weight of *sp*Mis18_1–120_, as measured using SEC‐MALS (size exclusion combined with multi‐angle light scattering), was 33,338 Da. Given that the calculated molecular weight of an *sp*Mis18_1–120_ monomer is 16,160 Da whereas a dimer would be 32,320 Da, this analysis independently confirms that *sp*Mis18_1–120_ forms a dimer in solution (Figs 2B and EV2A).

To identify the physiologically relevant dimer, we compared the extent of amino acid conservation of the interface residues and the buried surface area within the dimer I and dimer II interfaces. Dimer I is mainly stabilized by poorly conserved amino acid residues (F64, R66, L71, V73, S92, and I95 with the exception of R101) and involves 1,077 Å^2^ buried surface area. In contrast, dimer II is stabilized by highly conserved residues (V22, I31, Y114, and L116) and has an extensive binding interface (as compared to dimer I) with a 1,431 Å^2^ buried surface area. Based on this observation, we reasoned that dimer II is likely the physiologically relevant dimer. To test the basis of dimer formation, we generated several single point mutations at the dimer II interface (I31A, Y114A, Y114E, V22E) and determined whether they perturb the ability of *sp*Mis18_1–120_ to dimerize. In size‐exclusion chromatography (Fig 2B), recombinant *sp*Mis18_1–120_I31A mutant protein eluted later (Ve = 12.4 ml) than wild‐type *sp*Mis18_1–120_ (Ve = 11.8 ml), suggesting that this mutant protein forms a smaller entity than wild‐type. Further analysis of *sp*Mis18_1–120_I31A using SEC‐MALS confirmed that it is monomeric with a measured molecular weight of 15,955 Da (calculated molecular weight of a monomer is 16,160 Da) (Figs 2B and EV2A). Consistent with this, the other dimer II interface mutants Y114A, Y114E, and V22E also behaved as smaller entities compared to the wild‐type protein when analyzed by native PAGE (Fig EV2B), thus demonstrating that the dimerization of *sp*Mis18_1–120_ is mediated via the dimer II interface (Fig 2A).

### Yippee‐like globular domains of Mis18 proteins possess an intrinsic ability to form dimers

To test whether Yippee‐like globular domains of other Mis18 orthologs also display an intrinsic ability to form dimers, we expressed and purified recombinant fragments of human Mis18α (residues 77–187; *hs*Mis18α_77–187_) and Mis18β (56–183; *hs*Mis18β_56–183_) containing their Yippee‐like globular domains. SEC and SEC‐MALS analyses were conducted to assess their ability to form oligomers. While *hs*Mis18α_77–187_ eluted as a dimer at 15.5 ml in SEC with a measured molecular weight of 31,254 Da in SEC‐MALS (calculated MW of a dimer is 29,324 Da), the corresponding values for *hs*Mis18β_56‐183_ were 17.3 ml and 14,993 Da (calculated MW of a monomer is 13,591 Da), respectively, indicating that *hs*Mis18β_56–183_ exists as a monomer in solution (Fig 2C). We next tested whether *hs*Mis18α_77–187_ and *hs*Mis18β_56–183_ could interact to form a heterodimer, by mixing equimolar quantities of recombinant *hs*Mis18α_77–187_ and *hs*Mis18β_56–183_. In SEC, *hs*Mis18β_56–183_ co‐elutes with *hs*Mis18α_77–187_ at 15.4 ml suggesting that they form a heterodimer (Fig 2C). The molecular weight of this entity as measured by SEC‐MALS was 27,476 Da, confirming that *hs*Mis18α and *hs*Mis18β can heterodimerize through their respective Yippee‐like domains (Figs 2C and EV2C).

To test whether the mode of dimerization (dimer II mediated) is conserved from fission yeast to humans, we generated a homology model of the human *hs*Mis18α_77–187_–*hs*Mis18β_56–183_ heterodimer using the *sp*Mis18_1–120_ crystal structure described above as a template (Phyre2 server: http://www.sbg.bio.ic.ac.uk/phyre2/html/page.cgi?id=index) (Fig 2D). Our analysis of the modeled dimer interface (containing Mis18α residues Val 82, Arg 89, Pro 91, Asp 94, Tyr 176, and Leu 178, and Mis18β residues Val 77, His 84, Val 86, His 92, Tyr 172, Leu 174, Lys 175, and Thr 176) did not show any steric clashes and involved 1,387 Å^2^ buried surface area, similar to the *sp*Mis18_1–120_ dimer II interface. Moreover, mutations at the dimer interface (Mis18α V82E/Y176D and Mis18β V77E/Y172D) were sufficient to perturb the ability of *hs*Mis18α_77–187_ and *hs*Mis18β_56–183_ to form a heterodimer (Figs 2E and EV2D). This confirms that the Yippee‐like domains within Mis18 proteins employ a conserved mode of dimerization.

Structural and biochemical analyses of Yippee‐like domains from other proteins (Cereblon, RIG‐I and MsrB) have so far yielded no direct evidence that they possess an innate tendency to oligomerize *in vitro*. We therefore refer to the Yippee‐like domain of Mis18 orthologs as Mis eighteen Dimerization in Yippee (MeDiY) domain hereafter.

### The C‐terminal α‐helical domain induces tetramerization of *S*. *pombe* Mis18

**Figure EV3:**
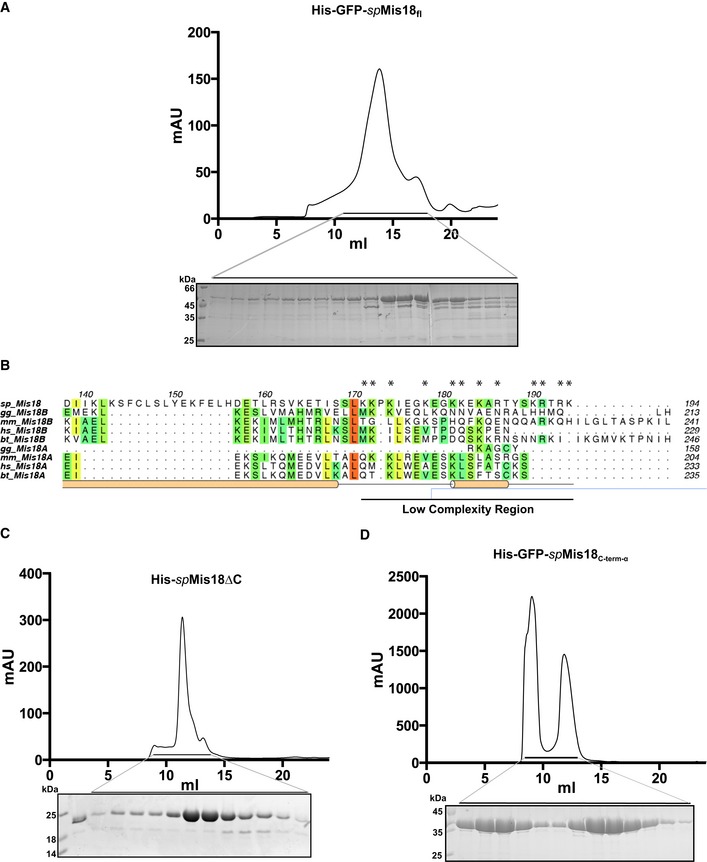
Characterization of the overall oligomeric state of *sp*Mis18_fl_ A
SEC profile and respective SDS–PAGE analysis of fractions (bottom panel) for His‐GFP‐*sp*Mis18_fl_. Superose 6 10/300 column was used for His‐GFP‐*sp*Mis18_fl_.BMultiple sequence alignment of *sp*Mis18 with its orthologs highlights the presence of a low‐complexity region (Lys/Arg‐rich region) at the extreme C‐terminus unique to *sp*Mis18.C, D
SEC profiles (top panels) and respective SDS–PAGE analyses of their fractions (bottom panels) for His‐*sp*Mis18ΔC and His‐GFP‐*sp*Mis18_C‐term‐α_, respectively. Superdex 200 increase 10/300 column was used for His‐*sp*Mis18ΔC and His‐GFP‐*sp*Mis18_C‐term‐α_.

**Figure 3:**
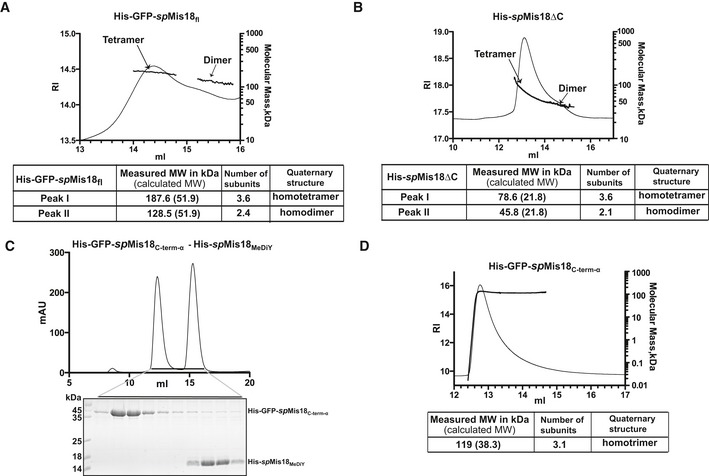
*sp*Mis18_fl_ forms a tetramer *in vitro* A, B
SEC‐MALS analysis of His‐GFP‐*sp*Mis18_fl_ and His‐*sp*Mis18ΔC, respectively. Refractive index (RI, left *y*‐axis) and molecular mass (kDa, right *y*‐axis) profiles show that both His‐GFP‐*sp*Mis18_fl_ (predicted MW of a monomer is 51.9 kDa) and His‐*sp*Mis18ΔC (predicted MW of a monomer 21.8 kDa) predominantly exist as tetramers (187.6 and 78.6 kDa, respectively) with a small population of dimer (128.5 and 45.8 kDa, respectively).C
SEC profile of a sample containing an equimolar mixture of purified His‐GFP‐*sp*Mis18_C‐term‐α_ and His‐*sp*Mis18_MeDiY_ (upper panel) and SDS–PAGE analysis of SEC fractions (bottom panel). His‐GFP‐*sp*Mis18_C‐term‐α_ and His‐*sp*Mis18_MeDiY_ eluted separately at 12.3 and 15.3 ml, respectively, demonstrating the inability of these domains to interact with each other.D
SEC‐MALS analysis of His‐GFP‐*sp*Mis18_C‐term‐α_ shows that it exists as a homotrimer with a measured molecular weight of 119.8 kDa (theoretically calculated MW of a monomer is 38.3 kDa).

In addition to the N‐terminal MeDiY domain, Mis18 orthologs possess a C‐terminal α‐helical domain *sp*Mis18_C‐term‐α_ (aa residues 121–end). To test whether *sp*Mis18_C‐term‐α_ can influence the overall oligomeric state of the protein, we purified recombinant full‐length *s*pMis18 (*sp*Mis18_fl_). Obtaining intact samples of *sp*Mis18_fl_ proved difficult either with or without His/His‐GFP tag, as it was sensitive to degradation from the C‐terminus (Fig EV3A). The stable partially degraded His‐GFP‐*sp*Mis18_fl_ when analyzed by SEC‐MALS appeared to form a tetramer (Fig 3A). Mass spectrometric peptide sequence coverage analysis of this sample revealed the loss of approximately 20 amino acids at the C‐terminus. Close examination of the amino acid sequence revealed the presence of a low‐complexity region at the extreme C‐terminus (amino acid residues 171–end, 15 out of 20 amino acid residues being Lys/Arg) that is unique to *sp*Mis18 (Fig EV3B). We therefore expressed and purified recombinant *sp*Mis18 with a C‐terminal truncation (*sp*Mis18ΔC; amino acids 1–168; Fig EV3C). SEC‐MALS analysis of *sp*Mis18ΔC revealed that it predominantly formed a tetramer (Fig 3B) and demonstrated a role for the α‐helical region downstream of the MeDiY domain (residues 121–168; Fig 1B) in *sp*Mis18 tetramerization. Since this α‐helical region is structurally conserved (based on secondary structure prediction; Fig 1B) between Mis18 proteins from evolutionarily distant eukaryotes, we propose that Mis18 oligomerization (either homo or hetero) mediated through the C‐terminus is also likely to be highly conserved.

### MeDiY and the C‐terminal α‐helical domain are independent structural modules

To obtain further insights into the overall architecture of the *sp*Mis18 oligomer, we tested whether *sp*Mis18_MeDiY_ and *sp*Mis18_C‐term‐α_ could interact with each other, or whether they existed as structurally independent modules. His‐*sp*Mis18_MeDiY_ and His‐GFP‐*sp*Mis18_C‐term‐α_ were purified individually and analyzed for complex formation using size‐exclusion chromatography. His‐GFP‐*sp*Mis18_C‐term‐α_ and His‐*sp*Mis18_MeDiY_ eluted separately at distinct elution volumes (12.3 and 15.3 ml, respectively). This demonstrates that the *sp*Mis18_MeDiY_ domain alone is unable to associate with *sp*Mis18_C‐term‐α_ (Figs 3C and EV3D).

We next tested whether *sp*Mis18_C‐term‐α_ can self‐associate to form oligomers. SEC‐MALS analysis of His‐GFP‐*sp*Mis18_C‐term‐α_ (untagged *sp*Mis18_C‐term‐α_ (8,775 Da) is too small for accurate mass detection) revealed that it is a homotrimer with a measured molecular weight of 119,886 Da (theoretical molecular weight of a monomer is 38,337 Da while that of a homotrimer would be 115,011 Da) (Fig 3D). We therefore conclude that *sp*Mis18 has two structurally independent domains, and they each possess the ability to homo‐oligomerize.

### Oligomerization of *sp*Mis18 via the MeDiY domain is required for its function

**Figure 4:**
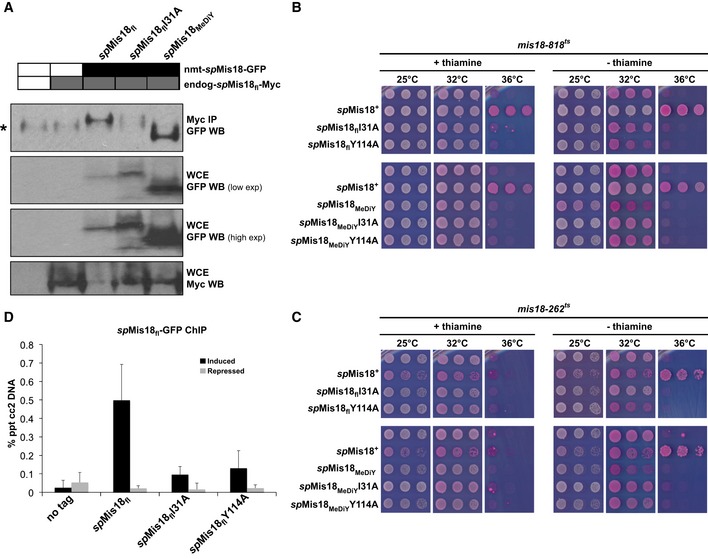
Dimerization mediated by the MeDiY domain promotes *sp*Mis18 function and centromere association A
*sp*Mis18_fl_‐Myc co‐immunoprecipitates with both GFP‐tagged *sp*Mis18_fl_ and *sp*Mis18_MeDiY_, but shows reduced association when MeDiY‐mediated dimerization is disrupted in *sp*Mis18_fl_I31A. The asterisk (*) in the top panel denotes the IgG heavy chain.B, CDimer II interface mutations I31A and Y114A affect the ability of ectopically expressed *sp*Mis18_fl_ to rescue the temperature sensitivity of *mis18‐818* and *mis18‐262* cells, while expression of *sp*Mis18_MeDiY_ alone confers a dominant‐negative effect on growth in a MeDiY dimerization‐dependent manner. Fivefold serial dilutions of cells expressing the indicated *sp*Mis18 constructs integrated at the *leu1* locus in the genome, spotted on complete PMG + phloxine B media supplemented with (repressed) or without (expressed) thiamine, and incubated at the indicated temperatures; dead cells stain dark pink.DMutations that disrupt MeDiY dimerization lead to reduced levels of *sp*Mis18_fl_ association with centromeres. qChIP analyses of *sp*Mis18_fl_‐GFP association with centromere 2 (cc2) in the indicated strains when grown in complete PMG media supplemented with (repressed) or without (expressed) thiamine. Error bars represent standard deviation between at least three biological replicates.

We next tested whether *sp*Mis18 dimerization is functionally important *in vivo*. Co‐immunoprecipitation assays were performed on *S. pombe* cells expressing endogenous *sp*Mis18_fl_ with a C‐terminal 8xMyc tag, and *sp*Mis18_fl_ or *sp*Mis18_fl_I31A expressed ectopically as a C‐terminally GFP‐tagged fusion protein. Endogenous wild‐type *sp*Mis18_fl_‐Myc associated efficiently with ectopic wild‐type *sp*Mis18_fl_‐GFP, confirming the interaction of *sp*Mis18_fl_ with itself *in vivo* (Fig 4A). Such self‐association of wild‐type *sp*Mis18 has also been demonstrated previously through yeast two‐hybrid assays 13, 15. In contrast, the *sp*Mis18_fl_I31A dimerization mutant showed significantly reduced association with endogenous *sp*Mis18_fl_‐Myc. Additionally, *sp*Mis18_MeDiY_ alone was sufficient to associate with *sp*Mis18_fl_‐Myc (Fig 4A). This highlights the role of *sp*Mis18_MeDiY_ in the overall oligomeric state of *sp*Mis18 *in vivo*.

To test whether *sp*Mis18_MeDiY_‐mediated oligomerization is essential for *sp*Mis18 function, we performed genetic complementation assays in which we evaluated the ability of dimerization mutants I31A and Y114A (in the context of both *sp*Mis18_fl_ and *sp*Mis18_MeDiY_), to rescue the temperature‐sensitive growth phenotype of *mis18‐818* (T49A) and *mis18‐262* (G117D) cells *in vivo* (Fig 4B and C) 10. Dimer‐disrupting mutants *sp*Mis18_fl_I31A and *sp*Mis18_fl_Y114A failed to rescue the viability defect at restrictive temperature in both *mis18‐262* and *mis18‐818* cells. Expression of *sp*Mis18_MeDiY_ alone, while failing to complement the loss of *sp*Mis18 function at 36°C, reproducibly conferred a dominant‐negative effect on growth in both *mis18‐262* and *mis18‐818* cells at 32°C. This inhibitory effect on growth of *mis18‐262* and *mis18‐818* cells depended on the ability of *sp*Mis18_MeDiY_ to dimerize, as the *sp*Mis18_MeDiY_I31A and Y114A mutations caused no such negative influence on growth (Fig 4B and C). We conclude that dimerization of *sp*Mis18 via the MeDiY domain is crucial for *sp*Mis18 function *in vivo*.

### Centromeric localization of *sp*Mis18 depends on MeDiY‐mediated dimerization

**Figure EV4:**
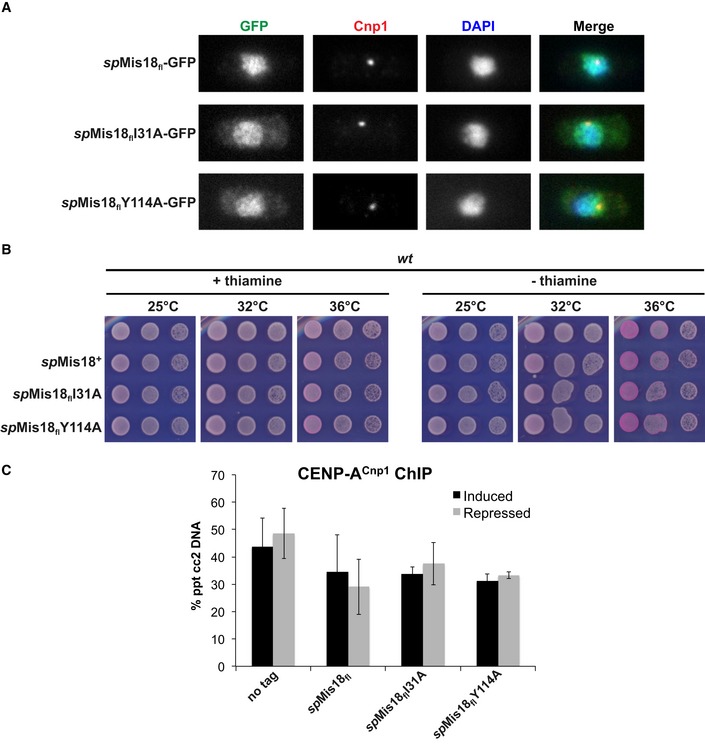
Ectopically expressed *sp*Mis18_fl_ proteins localize to the nucleus and do not alter cell growth Ectopically expressed *sp*Mis18_fl_‐GFP localizes throughout the nucleus in a MeDiY dimerization‐independent manner. Immunofluorescence of wild‐type *S. pombe* cells ectopically expressing GFP‐tagged *sp*Mis18_fl_ (wt or mutants), stained with antibodies to GFP (green) and CENP‐A^C^ ^np1^ (red), and DAPI (blue).Ectopic expression of dimer II interface mutants does not affect growth of wild‐type *S. pombe* cells. Fivefold serial dilutions of cells expressing the indicated *sp*Mis18_fl_ constructs integrated at the *leu1* locus in the genome, spotted on complete PMG + phloxine B media supplemented with (repressed) or without (expressed) thiamine, and incubated at the indicated temperatures; dead cells stain dark pink.Mutations that disrupt MeDiY dimerization cause no significant change in CENP‐A^C^ ^np1^ association with centromeres in wild‐type cells. qChIP analyses of CENP‐A^C^ ^np1^ association with centromere 2 (cc2) in the indicated strains when grown in complete PMG media supplemented with (repressed) or without (expressed) thiamine. Error bars represent standard deviation between at least three biological replicates.

*sp*Mis18 is known to associate with centromeres, where it is required for the incorporation of CENP‐A^Cnp1^ 10. Chromatin immunoprecipitation analyses were performed to determine whether dimerization mediated by the MeDiY domain is required for the association of *sp*Mis18_fl_ with centromeres. Disruption of *sp*Mis18 MeDiY‐mediated dimerization through *sp*Mis18_fl_I31A or *sp*Mis18_fl_Y114A mutations resulted in reduced association of ectopically expressed *sp*Mis18_fl_‐GFP with centromeres in wild‐type cells (Fig 4D), although no significant defects in subcellular localization of *sp*Mis18_fl_‐GFP were observed (Fig EV4A). Additionally, cell growth and CENP‐A^Cnp1^ association with centromeres were essentially unaffected in these cells (Fig EV4B and C). Thus, MeDiY domain‐mediated dimerization ensures optimal *sp*Mis18 association with centromeres.

Centromeric association of the Mis18 complex is one of the early key steps involved in the establishment of CENP‐A nucleosomes at centromeres 17. Although some of the components of the Mis18 complex show variations among different species (particularly the absence of Mis18BP1/KNL2 in fission yeast), the function of the Mis18 complex in targeting HJURP to centromeres is well conserved 14, 15, 26, 27, 28. The molecular mechanism by which the Mis18 complex is targeted to centromeres, however, remains to be determined, and any structural insights into the molecular architecture of the Mis18 complex and/or its components have the potential to advance our understanding of centromere establishment.

The Mis18 complex is critical for CENP‐A deposition at centromeres; however, Mis18 is poorly characterized in terms of its structure and function. Here, we show that fission yeast Mis18 and its human isoforms, Mis18α and Mis18β, possess two structurally distinct domains: an N‐terminal “Yippee‐like” globular domain and a C‐terminal α‐helical domain. Structural alignments revealed unambiguous structural homology between the N‐terminal “Yippee‐like” globular domain and proteins of diverse function such as Cereblon 21, RIG‐I 22, MSS4 23, and MsrB 24, as well as identified a conserved substrate‐binding pocket in Mis18 required for its function. However, predicting the identity and nature of Mis18 binding partner(s) seems non‐trivial, as the amino acids that make up the binding pocket and their spatial orientation are markedly different among the various homologous proteins.

A key finding of our analyses is the ability of the “Yippee‐like” globular domain (MeDiY) of *S. pombe* and human Mis18 proteins to dimerize. To date, none of the other related domains are known to form dimers. Interestingly, *S. pombe* Mis18 and also human Mis18 29, in the presence of Mis18_C‐term‐α_, forms a tetramer. Notably, ectopic expression of the MeDiY domain alone, while failing to complement the loss of *sp*Mis18 function conferred a dominant‐negative effect on the growth of *mis18* mutants, implicating both MeDiY and C‐terminal α‐helical domains in ensuring the required oligomeric structure for function. Specifically, perturbing the dimeric interface of the MeDiY domain affects the centromere localization of *sp*Mis18. Identifying the binding partners of MeDiY and determining how (and if) Mis18 oligomerization can influence substrate recognition are important questions to be addressed in the future to unravel the molecular mechanisms that mediate centromere localization and function of the Mis18 complex. A previous study has shown that HJURP dimerization is required for stable deposition of CENP‐A nucleosomes at centromeres and implicated a direct role for HJURP dimerization in forming octameric CENP‐A nucleosomes 30. These observations highlight protein oligomerization‐mediated regulation as an emerging regulatory theme for the inherently complex process of centromere establishment and maintenance.

## Materials and Methods

### Expression and purification of recombinant *S. pombe* proteins

*Schizosaccharomyces pombe* Mis18_fl_, Mis18_MeDiY_, Mis18ΔC, and Mis18_C‐term‐α_ codon‐optimized sequences (GeneArt) were cloned into pEC‐K‐3C‐His or 9GFP (Addgene) LIC vectors with N‐terminal His or His‐GFP tags cleavable with either a 3C or TEV site, respectively. Mutations were introduced using QuikChange site‐directed mutagenesis protocol (Stratagene). All proteins were expressed using *E. coli* BL21 Gold; His‐*sp*Mis18_MeDiY_ was grown in Super Broth and induced for 6 h at 25°C using 0.3 mM IPTG. His‐*sp*Mis18ΔC was grown in Super Broth, while His‐GFP *sp*Mis18_C‐term‐α_ and His‐GFP‐*sp*Mis18_fl_ were grown in 2× TY before inducing for 16 h at 18°C by adding 0.3 mM IPTG.

All proteins were lysed via sonication in a lysis buffer containing 20 mM Tris pH 8 (or 8.5 for His‐*sp*Mis18_MeDiY_), 35 mM imidazole and 2 mM BME supplemented with 10 μg/ml DNase, 1 mM PMSF, and complete EDTA‐free (Roche). The following NaCl concentrations were used in the lysis buffer: 500 mM for His‐*sp*Mis18_MeDiY_, 50 mM for His‐*sp*Mis18ΔC, and 100 mM for His‐GFP‐*sp*Mis18_C‐term‐α_ or His‐GFP‐*sp*Mis18_fl_. After clarification, His‐GFP‐*sp*Mis18_C‐term‐α_, His‐GFP‐*sp*Mis18_fl_, and His‐*sp*Mis18_MeDiY_ proteins were purified using a 5‐ml HisTrap HP column (GE Healthcare), while His‐*sp*Mis18ΔC was purified by batch mode using HisPur Ni‐NTA resin (Thermo Scientific). Resin was washed with lysis buffer, and then, His‐*sp*Mis18_1–120,_ His‐GFP‐*sp*Mis18_C‐term‐α_ or His‐GFP‐*sp*Mis18_fl_ received additional washes with 20 mM Tris pH 8/8.5, 35 mM imidazole, 50 mM KCl, 10 mM MgCl_2_, 2 mM ATP, and 2 mM BME. The following NaCl concentrations were used: 500 mM for His‐*sp*Mis18_MeDiY_ or 1 M for His‐GFP‐*sp*Mis18_C‐term‐α_ and His‐GFP‐*sp*Mis18_fl_. Proteins were finally washed in lysis buffer and then eluted with 20 mM Tris pH 8/8.5, 500 mM imidazole, and 2 mM BME. The following NaCl concentrations were used in the elution buffer: 500 mM for His‐*sp*Mis18_MeDiY_, His‐*sp*Mis18ΔC, and His‐GFP‐*sp*Mis18_C‐term‐α_, and 100 mM NaCl for His‐GFP‐*sp*Mis18_fl_. Eluted His‐*sp*Mis18_1–120_ was used directly for crystallization trial, while all other proteins were subjected to size‐exclusion chromatography. His‐*sp*Mis18ΔC was loaded onto Superdex 200 Hi‐load 16/600 (GE Healthcare) equilibrated with 20 mM Tris pH 8, 400 mM NaCl, and 1 mM TCEP. Appropriate fractions were pooled and injected onto a Superdex 200 increase 10/300 column (GE Healthcare) equilibrated with 20 mM Tris pH 8, 50 mM NaCl, and 4 mM DTT. His‐GFP‐*sp*Mis18_C‐term‐α_ was applied to a Superdex 200 increase 10/300 column equilibrated with 20 mM Tris pH 8, 150 mM NaCl, and 2 mM DTT. His‐GFP‐*sp*Mis18_fl_ was applied to a Superose 6 10/300 column (GE Healthcare) equilibrated with 20 mM Tris pH 8, 300 mM NaCl, and 2 mM DTT. Fractions were analyzed on SDS–PAGE and stained with Coomassie blue.

### Expression and purification of recombinant human proteins

Human Mis18α_fl_, Mis18α_77–187_, Mis18β_fl_, and Mis18β_56–183_ codon‐optimized sequences (GeneArt) were cloned into pEC‐K‐3C‐His LIC vector or pGEX‐6P‐1 (GE Healthcare). Human proteins were expressed separately or co‐expressed using pGEX‐6P‐1 and pEC‐K‐3C‐His in BL21 Gold in a similar manner to *sp*Mis18ΔC. His‐*hs*Mis18α_77–187_ was lysed via sonication in 20 mM Tris pH 8, 500 mM NaCl, 35 mM imidazole, and 4 mM BME with 10 μg/ml DNase, 1 mM PMSF, and complete EDTA‐free. Clarified lysate was loaded onto a 5‐ml HisTrap HP column and washed with lysis buffer and then with 20 mM Tris pH 8, 1 M NaCl, 35 mM imidazole, 50 mM KCl, 10 mM MgCl_2_, 2 mM ATP, and 4 mM BME and re‐equilibrated in lysis buffer before eluting with 20 mM Tris pH 8, 500 mM NaCl, 500 mM imidazole, and 4 mM BME. GST‐*hs*Mis18β_56–183_ was sonicated in 20 mM Tris pH 8, 500 mM NaCl, and 4 mM DTT with 10 μg/ml DNase, 1 mM PMSF, and complete EDTA‐free. Cleared lysate was loaded on 12 ml glutathione–Sepharose (GE Healthcare) in batch mode and washed in lysis buffer and then with 20 mM Tris pH 8, 1 M NaCl, 50 mM KCl, 10 mM MgCl_2_, 2 mM ATP, and 4 mM DTT followed by lysis buffer. 3C protease was added to cleave the tag on the beads overnight and untagged protein collected. To obtain the *hs*Mis18α_77–187_/Mis18β_56–183_ complex, proteins were co‐expressed and purified as described for GST‐*hs*Mis18β_56–183_. All proteins were separated on Superose 6 10/300 pre‐equilibrated with 20 mM Tris pH 8, 200 mM NaCl, and 5 mM DTT.

### His/GST pull‐down assays

To test for protein–protein interactions, purified His‐tagged *sp*Mis18_MeDiY_ and His‐GFP‐tagged *sp*Mis18_C‐term‐α_ proteins were mixed and applied to a Superdex 200 increase 10/300 column equilibrated with 20 mM Tris pH 8, 150 mM NaCl, and 2 mM DTT and fractions analyzed via SDS–PAGE. To test for interaction of human *hs*Mis18α and β, 10 ml of culture of co‐expressed proteins was grown and sonicated in 20 mM Tris pH 8, 100 mM NaCl, and 2 mM BME. After clarification, supernatants were split and 35 mM imidazole added to half before incubation for 1 h with either glutathione–Sepharose or HisPur Ni‐NTA resin and then washed with buffer. Purified proteins were run on SDS–PAGE.

### Native PAGE

12% native PAGE gels (22 mM Tris pH 8.8, 12% acrylamide with stacking 320 mM Tris pH 8.8, 4% acrylamide) were run in 25 mM Tris and 192 mM glycine at 150 V for 30 mins. Proteins in 1× sample buffer (31.25 mM Tris pH 6.8, 12.5% glycerol, 0.5% bromophenol blue) and Native Marker (Life Technologies) were loaded and then run at 150 V for 3 h.

### Crystallization and data collection

Crystallization trials were performed using a nanoliter crystallization robot at the Edinburgh crystallization facility. Crystals were grown by vapor diffusion method. Diffraction quality crystals were obtained using well buffer containing 0.2 M ammonium chloride/formate/acetate/phosphate and 20% PEG 3350 (with the measured pH of the solution in the range of 6.2–8). 15–20 mg/ml protein sample was mixed with the well buffer in a 1:1 ratio. Crystals were briefly transferred to cryoprotectant solution (crystallization solutions were supplemented with glycerol or ethylene glycol to a final concentration 25%) before flash cooling in liquid nitrogen. The crystals diffracted to 2.6 Å resolution at the MX beamlines of the Diamond Light Source (Table 1).

### Crystal structure solution and refinement

The structure of *sp*Mis18_MeDiY_ was determined by the single anomalous dispersion (SAD) method using the anomalous signal of intrinsically bound Zn^2+^ ion. Data were processed using XIA2 and scaled with SCALA of CCP4 31. SAD phasing and the calculation of the initial map were performed using phenix.autosol from the PHENIX suite of programs 32. The model was built by iterative rounds of manual building with COOT 33, and refinement was done using Refmac5 of CCP4. Data collection, phasing, and refinement statistics are shown in Table 1.

### SEC‐MALS

Size‐exclusion chromatography coupled to multi‐angle light scattering (SEC‐MALS) was performed at the Edinburgh Protein Production Facility at room temperature using a ÄKTA FPLC. 100 μl of protein at 1 mg/ml was loaded onto either Superose 6 10/300, Superdex 75 10/300, or Superdex 200 10/300 columns (GE Healthcare) pre‐equilibrated with 50 mM HEPES pH 8, 150 mM NaCl, and 5 mM DTT for His‐*sp*Mis18_MeDiY_, His‐GFP‐*sp*Mis18_C‐term‐α_, and *hs*Mis18α_77–187_/*hs*Mis18β_56–183_, or 50 mM HEPES pH 8, 50 mM NaCl, and 5 mM DTT for His‐*sp*Mis18ΔC or 50 mM HEPES pH 8, 300 mM NaCl, and 1 mM TCEP for His‐GFP *sp*Mis18_fl_. A MiniDAWN in‐line detector (Wyatt Technology) was used to measure MALS, while a Viscotek RI Detector (Wyatt Technology) was used to detect refractive index. Data were analyzed using ASTRA^™^ software (Wyatt Technology).

### Plasmids and *S. pombe* strains

For *in vivo* assays in *S. pombe*,* sp*Mis18 cDNA (wt, I31A, Y114A, Y90A or Y74A Y90A T105A S107K in the context of Mis18_fl_ or Mis18_MeDiY_) was PCR‐amplified and cloned into the pDUAL‐GFH41 vector, which allows for expression of C‐terminally GFP‐tagged *sp*Mis18 (wt or mutants) under the control of the medium‐strength *nmt41* promoter that is induced in the absence of thiamine in culture media 34. *sp*Mis18 constructs cloned into the pDUAL‐GFH41 vector were then either transformed as such and selected in the absence of uracil in growth media, or integrated into the genome at the *leu1* locus, in wt, *mis18‐818,* or *mis18‐262* cells. Genotypes of *S. pombe* strains used in this study are listed in Table EV1.

### Genetic complementation assays

Fivefold serial dilutions of *mis18‐262, mis18‐818,* or wt cells expressing GFP‐tagged *sp*Mis18_fl_ or *sp*Mis18_MeDiY_ (wt or mutants) either from an ectopic pDUAL‐GFH41 plasmid (selected for in media lacking uracil; Fig 1E) or from the *leu1* locus (integrated in the genome; Figs 4B and C and EV4B), were spotted onto PMG media containing phloxine B supplemented with or without thiamine and incubated at the indicated temperatures for 3–5 days.

### Co‐immunoprecipitation and Western analyses

For co‐immunoprecipitation experiments, cells expressing endogenous *sp*Mis18_fl_‐Myc and ectopic *sp*Mis18‐GFP (wt or mutants; from the *leu1* locus) were cultured in complete PMG media lacking thiamine for 21 h and processed as previously described 11. Immunoprecipitation was performed using rabbit anti‐myc antibody A14 (Santa Cruz Biotech) and Western analyses using monoclonal anti‐GFP (Roche) or anti‐myc 9B11 (Cell Signaling).

### Quantitative chromatin immunoprecipitation

Anti‐GFP ChIP, anti‐CENP‐A^Cnp1^ ChIP, and real‐time PCR analyses were performed as previously described 11, on wild‐type *S. pombe* cells expressing ectopic GFP‐tagged *sp*Mis18_fl_ (wt or mutants; from the *leu1* locus) cultured in complete PMG media lacking thiamine for 21 h.

### Cytology

Immunolocalization and microscopy were performed as previously described 11 on wild‐type *S. pombe* cells expressing ectopic GFP‐tagged *sp*Mis18_fl_ (wt or mutants; from the *leu1* locus) cultured in complete PMG media lacking thiamine for 21 h.

### Data deposition

Atomic coordinates of the structure and structure factors are deposited in the RCSB protein data bank (www.rcsb.org) with accession code 5HJ0.

## Author contributions

LS, BM‐P, RB, FS, RK‐S, GR, and AAJ performed experiments and analyzed data. LS, BM‐P, RCA, and AAJ designed experiments, analyzed data, and wrote the manuscript.

## Conflict of interest

The authors declare that they have no conflict of interest.

## Supporting information



Expanded View Figures PDF

Table EV1

Review Process File
