# Supplementary material for: Centromere localization and function of Mis18 requires Yippee‐like domain‐mediated oligomerization
Source: EMBO Rep. 2016 Mar 3;17(4):496–507. doi: 10.15252/embr.201541520 (PMC4818781; doi:10.15252/embr.201541520)
Supplement: Supplementary file 1 — Expanded View Figures PDF [file EMBR-17-496-s001.pdf]

Expanded View Figures

Figure EV1. Structural characterization of the N-terminal Yippee-like globular domain of *sp*Mis18.

A Stereo image of electron density map ($2F_o$–F_c, contoured at 1σ) corresponding to β-sheet I.

B Structural superposition of *sp*Mis18$_{1–120}$ onto its closest structural homologs, Cereblon TBD (PDB: 4TZC), RIG-I (PDB: 2QFD), Mss4(PDD: 1FWQ), and MsrB (PDB: 3E0O). Structural superpositions were carried out using PDBefold web server (http://www.ebi.ac.uk/msd-srv/ssm).

C Structural superposition of *sp*Mis18$_{1–120}$ with its structural homologs bound to their substrates. For clarity, structural homologs of *sp*Mis18$_{1–120}$ are not shown.

D Surface representation of *sp*Mis18$_{1–120}$ where the cradle-shaped pocket and amino acid residues mutated in the complementation assay (Fig 1E) are highlighted.

 © 2016 The Authors

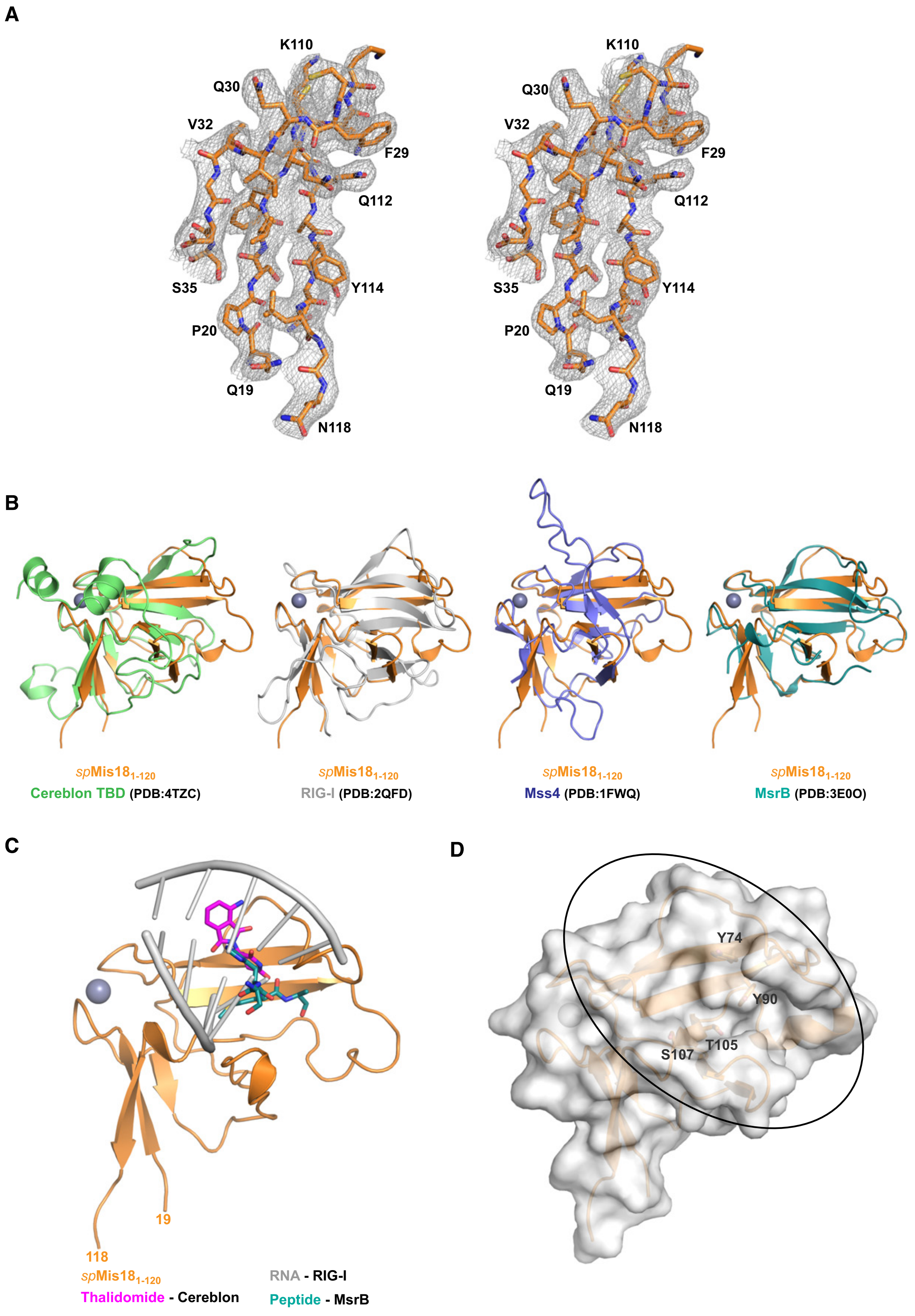
A
K110
Q30
V32
F29
Q112
S35
Y114
P20
Q19
N118
K110
Q30
V32
F29
Q112
S35
Y114
P20
Q19
N118
B
spMis18 1-120
Cereblon TBD (PDB:4TZC)
spMis18 1-120
RIG-I (PDB:2QFD)
spMis18 1-120
Mss4 (PDB:1FWQ)
spMis18 1-120
MsrB (PDB:3E0O)
C
19
118
spMis18 1-120
Thalidomide - Cereblon
RNA - RIG-I
Peptide - MsrB
D
Y74
Y90
T105
S107

Figure EV1.

Figure EV2. Evaluation of the dimerization ability of Yippee-like domains of Mis18 proteins.

A SEC-MALS profiles of His-*sp*Mis18$_{1–120}$ (left panel) and His-*sp*Mis18$_{1–120}$I31A (right panel). Refractive index (RI, left *y*-axis) and molar mass (MW, right *y*-axis) profiles show that His-*sp*Mis18$_{1–120}$ (predicted MW of a monomer: 16.2 kDa) is a dimer (measured MW: 33.3 kDa) and His-*sp*Mis18$_{1–120}$I31A (predicted MW of monomer: 16.2 kDa) is a monomer (measured MW: 15.6 kDa).

B Native PAGE analysis of dimer interface II mutants. Interface mutants I31A, V22E, Y114E, and Y114A migrated faster than wt *sp*Mis18$_{1–120}$, confirming the effect these mutations have on the oligomeric structure of *sp*Mis18$_{1–120}$.

C SEC-MALS profiles of His-*hs*Mis18$\alpha_{77–187}$ (top panel), His-*hs*Mis18$\beta_{56–183}$ (middle panel), and His-*hs*Mis18$\alpha_{77–187}$–His-*hs*Mis18$\beta_{56–183}$ (bottom panel). While His-*hs*Mis18$\alpha_{77–187}$ (predicted MW of a monomer: 14.7 kDa) formed a homodimer (measured MW: 31.3 kDa), His-*hs*Mis18$\beta_{56–183}$ (predicted MW of a monomer: 13.6 kDa) remained as a monomer (measured MW: 15.0 kDa). His-*hs*Mis18$\alpha_{77–187}$–His-*hs*Mis18$\beta_{56–183}$ (predicted MW of a monomer: 28.3 kDa) formed a heterodimer (measured MW: 27.5 kDa).

D SDS–PAGE analysis of Ni-NTA pull-down experiment, where His-*hs*Mis18$\alpha_{77–187}$ and GST-*hs*Mis18$\beta_{56–183}$ were co-expressed in *E. coli* as wt or dimer-disrupting mutant proteins. While the GST pull-down assay shown in Fig 2E confirmed the inability of GST-*hs*Mis18$\beta_{56–183}$V77E/Y172D to interact with His-*hs*Mis18$\alpha_{77–187}$V82E/Y176D, the corresponding Ni-NTA pull-down shown here confirms the abundant expression of His-*hs*Mis18$\alpha_{77–187}$V82E/Y176D in the input. We note that His-*hs*Mis18$\alpha_{77–187}$V82E/Y176D migrates as a doublet. Western blot analysis (data not shown) using anti-His antibody confirmed the presence of His-tag on both bands suggesting the unstable nature of His-*hs*Mis18$\alpha_{77–187}$V82E/Y176D mutant (possibly due to its inability to homo- or heterodimerize).

© 2016 The Authors

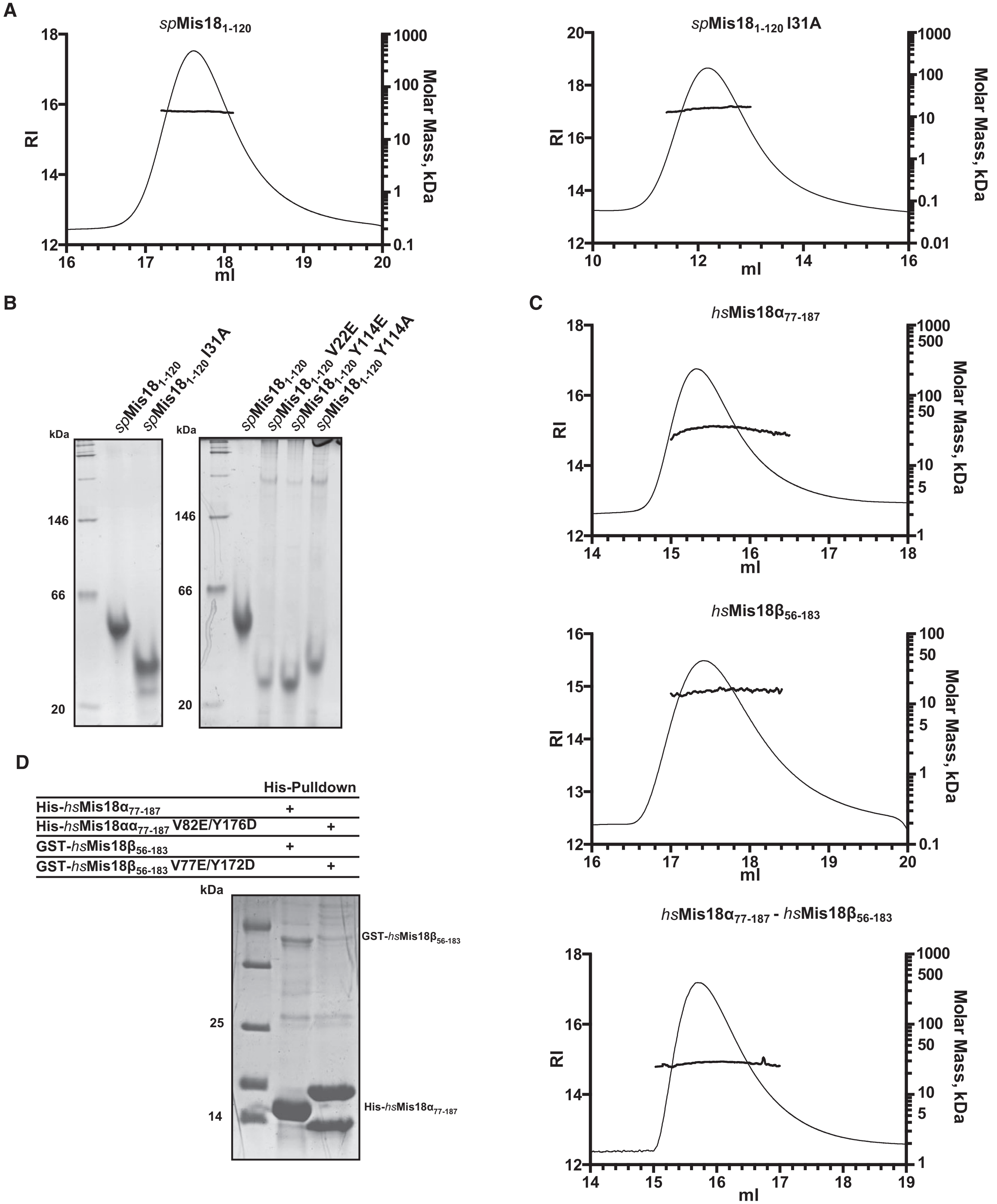
A
spMis18 1-120
RI
Molar Mass, kDa
ml
spMis18 1-120 I31A
B
spMis18 1-120
spMis18 1-120 I31A
spMis18 1-120 V22E
spMis18 1-120 Y114E
spMis18 1-120 Y114A
kDa
146
66
20
C
hsMis18α 77-187
hsMis18β 56-183
hsMis18α 77-187 - hsMis18β 56-183
D
His-Pulldown
His-hsMis18α 77-187
His-hsMis18αα 77-187 V82E/Y176D
GST-hsMis18β 56-183
GST-hsMis18β 56-183 V77E/Y172D
25
14
GST-hsMis18β 56-183
His-hsMis18α 77-187

Figure EV2.

Figure EV3. Characterization of the overall oligomeric state of *sp*Mis18$_{fl}$.

A SEC profile and respective SDS–PAGE analysis of fractions (bottom panel) for His-GFP-*sp*Mis18$_{fl}$. Superose 6 10/300 column was used for His-GFP-*sp*Mis18$_{fl}$.

B Multiple sequence alignment of *sp*Mis18 with its orthologs highlights the presence of a low-complexity region (Lys/Arg-rich region) at the extreme C-terminus unique to *sp*Mis18.

C, D SEC profiles (top panels) and respective SDS–PAGE analyses of their fractions (bottom panels) for His-*sp*Mis18ΔC and His-GFP-*sp*Mis18$_{C\text{-}term\text{-}\alpha}$, respectively. Superdex 200 increase 10/300 column was used for His-*sp*Mis18ΔC and His-GFP-*sp*Mis18$_{C\text{-}term\text{-}\alpha}$.

© 2016 The Authors

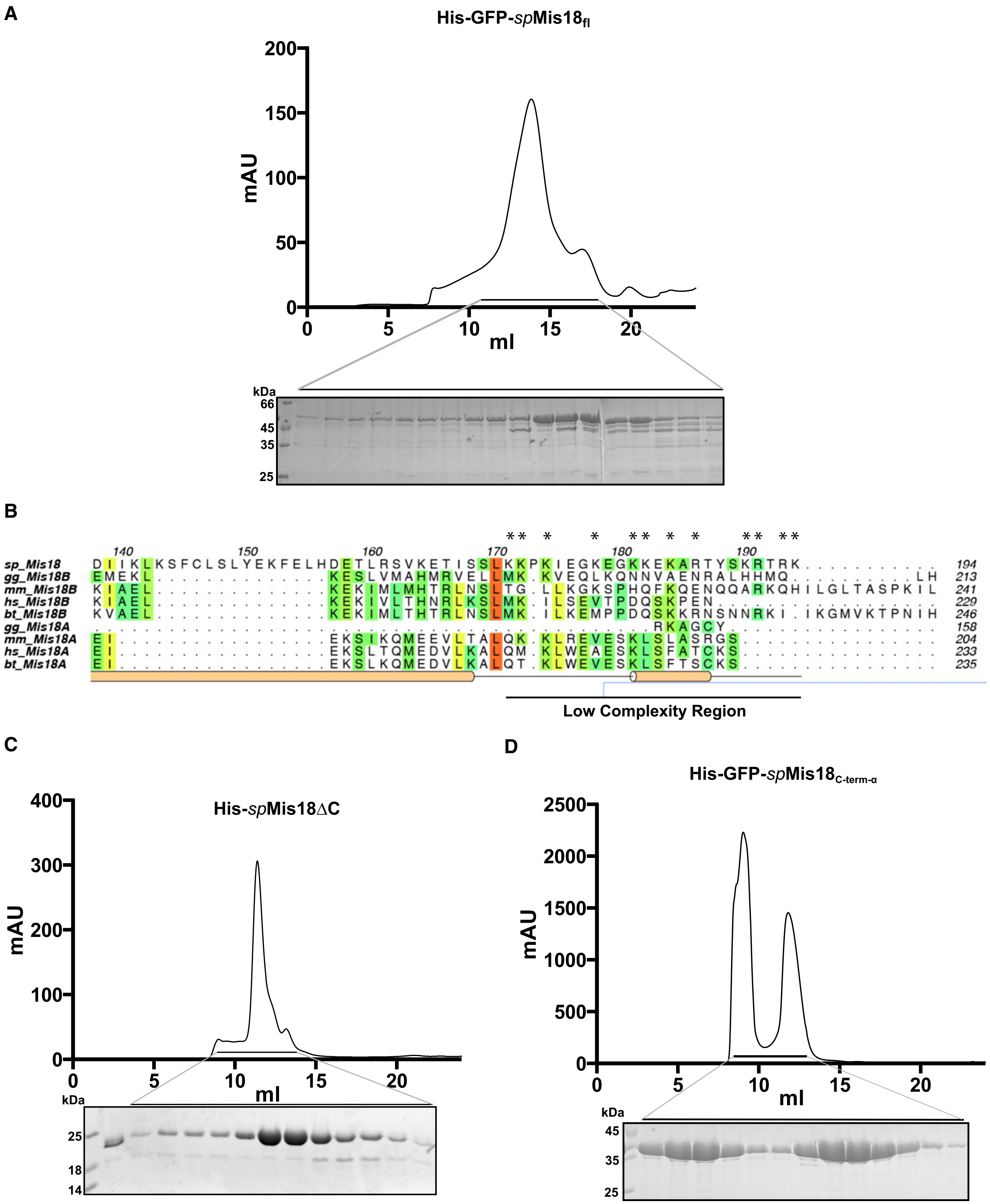
A
His-GFP-spMis18fl
mAU
200
150
100
50
0
0 5 10 15 20
ml
kDa
66
45
35
25
B
** * * ** * * ** **
140 150 160 170 180 190
sp_Mis18 DIIKLKSFCLSLYEKFELHDETLRSVKETISSLKKPKIEGKEGKKEKARTYSKRTRK… 194
gg_Mis18B EMEKL…KESLVMAHMRVELLMK.KVEQLKQNNVAENRALHHMQ…LH 213
mm_Mis18B KIAEL…KEKIMLMHTRLNSLTG.LLKGKSPHQFKQENQQARKQHILGLTASPKIL 241
hs_Mis18B KIAEL…KEKIVLTHNRLKSLMK.ILSEVTPDQSKPEN… 229
bt_Mis18B KVAEL…KEKIMLTHTRLNSLMK.ILKEMPPDQSKKRNSNNRKI.IKGMVKTPNIH 246
gg_Mis18A …RKAGCY… 158
mm_Mis18A EI…EKSIKQMEEVLTALQK.KLREVESKLSLASRGS… 204
hs_Mis18A EI…EKSLTQMEDVLKALQM.KLWEAESKLSFATCKS… 233
bt_Mis18A EI…EKSLKQMEDVLKALQT.KLWEVESKLSFTSCKS… 235
Low Complexity Region
C
His-spMis18ΔC
mAU
400
300
200
100
0
0 5 10 15 20
ml
kDa
25
18
14
D
His-GFP-spMis18C-term-α
mAU
2500
2000
1500
1000
500
0
0 5 10 15 20
ml
kDa
45
35
25

Figure EV3.

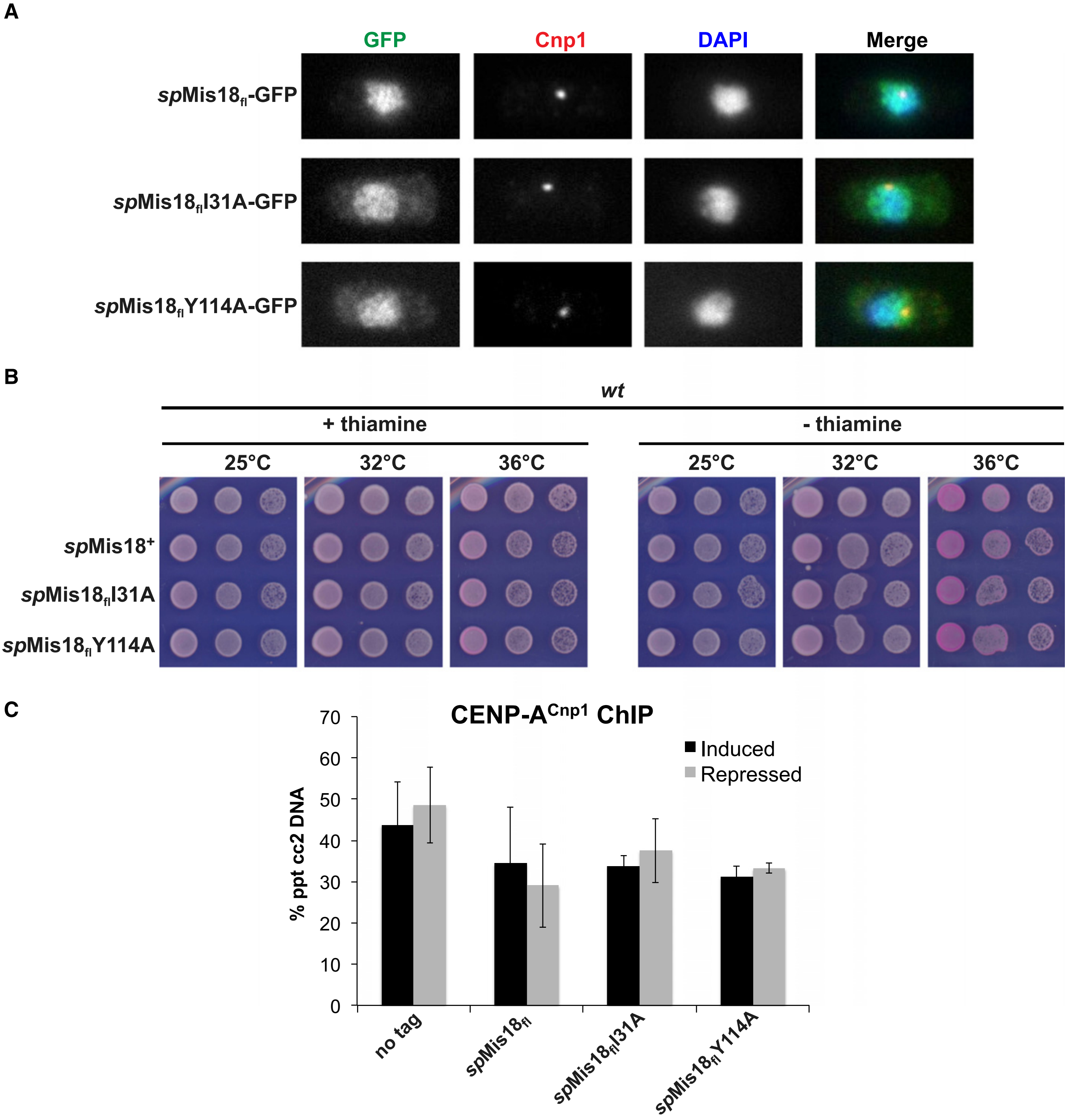

Figure EV4. Ectopically expressed *sp*Mis18fl proteins localize to the nucleus and do not alter cell growth.

A Ectopically expressed *sp*Mis18fl-GFP localizes throughout the nucleus in a MeDiY dimerization-independent manner. Immunofluorescence of wild-type *S. pombe* cells ectopically expressing GFP-tagged *sp*Mis18fl (wt or mutants), stained with antibodies to GFP (green) and CENP-A^{Cnp1} (red), and DAPI (blue).

B Ectopic expression of dimer II interface mutants does not affect growth of wild-type *S. pombe* cells. Fivefold serial dilutions of cells expressing the indicated *sp*Mis18fl constructs integrated at the *leu1* locus in the genome, spotted on complete PMG + phloxine B media supplemented with (repressed) or without (expressed) thiamine, and incubated at the indicated temperatures; dead cells stain dark pink.

C Mutations that disrupt MeDiY dimerization cause no significant change in CENP-A^{Cnp1} association with centromeres in wild-type cells. qChIP analyses of CENP-A^{Cnp1} association with centromere 2 (cc2) in the indicated strains when grown in complete PMG media supplemented with (repressed) or without (expressed) thiamine. Error bars represent standard deviation between at least three biological replicates.

 © 2016 The Authors
