# Supplementary material for: Centromere localization and function of Mis18 requires Yippee‐like domain‐mediated oligomerization
Source: EMBO Rep. 2016 Mar 3;17(4):496–507. doi: 10.15252/embr.201541520 (PMC4818781; doi:10.15252/embr.201541520)
Supplement: Supplementary file 2 — Table EV1 [file EMBR-17-496-s002.doc]

**Table EV1: *S. pombe* strains used in this study**

| **Strain** | **Figure** | **Genotype** |
| --- | --- | --- |
| B864 | 1E | *h^+^ leu1-32 ura4-D18 ade6-M210 his3-D1 arg3-D4 mis18-262 // pDUAL-GFH41 pld* |
| B863 | 1E | *h^+^ leu1-32 ura4-D18 ade6-M210 his3-D1 arg3-D4 mis18-262 // mis18^+^:pDUAL-GFH41 pld* |
| B921 | 1E | *h^+^ leu1-32 ura4-D18 ade6-M210 his3-D1 arg3-D4 mis18-262 // mis18 (Y90A):pDUAL-GFH41 pld* |
| LS723 | 1E | *h^+^ leu1-32 ura4-D18 ade6-M210 his3-D1 arg3-D4 mis18-262 // mis18 (Y74A Y90A T105A S107K):pDUAL-GFH41 pld* |
| 1645 | 4A, 4D, S4 | *h^+^ leu1-32 ura4-D18 ade6-M210 his3-D1 arg3-D4* |
| A9711 | 4A | *h^-^ leu1-32 ura4-D18 ade6? his3? arg3? mis18:myc-ura4+* |
| B889 | 4A | *h? leu1-32 ura4-D18 ade6? his3? arg3? mis18:myc-ura4+ // leu1::mis18^+^:pDUAL-GFH41* |
| B891 | 4A | *h? leu1-32 ura4-D18 ade6? his3? arg3? mis18:myc-ura4+ // leu1::mis18 (I31A):pDUAL-GFH41* |
| B897 | 4A | *h? leu1-32 ura4-D18 ade6? his3? arg3? mis18:myc-ura4+ // leu1::mis18_MeDiY_:pDUAL-GFH41* |
| B789 | 4B | *h^+^ leu1 ura4 his2 mis18-818* |
| B964 | 4B | *h^+^ leu1-32 ura4-D18 his2 mis18-818 // leu1::mis18^+^:pDUAL-GFH41* |
| B967 | 4B | *h^+^ leu1-32 ura4-D18 his2 mis18-818 // leu1::mis18 (I31A):pDUAL-GFH41* |
| B972 | 4B | *h^+^ leu1-32 ura4-D18 his2 mis18-818 // leu1::mis18 (Y114A):pDUAL-GFH41* |
| B973 | 4B | *h^+^ leu1-32 ura4-D18 his2 mis18-818 // leu1:: mis18_MeDiY_:pDUAL-GFH41* |
| B976 | 4B | *h^+^ leu1-32 ura4-D18 his2 mis18-818 // leu1:: mis18_MeDiY_(I31A):pDUAL-GFH41* |
| B980 | 4B | *h^+^ leu1-32 ura4-D18 his2 mis18-818 // leu1:: mis18_MeDiY_(Y114A):pDUAL-GFH41* |
| A2377 | 4C | *h^+^ leu1-32 ura4-D18 ade6-M210 his3-D1 arg3-D4 mis18-262* |
| B958 | 4C | *h^+^ leu1-32 ura4-D18 ade6-M210 his3-D1 arg3-D4 mis18-262 // leu1::mis18^+^:pDUAL-GFH41* |
| B883 | 4C | *h^+^ leu1-32 ura4-D18 ade6-M210 his3-D1 arg3-D4 mis18-262 // leu1::mis18 (I31A):pDUAL-GFH41* |
| B959 | 4C | *h^+^ leu1-32 ura4-D18 ade6-M210 his3-D1 arg3-D4 mis18-262 // leu1::mis18 (Y114A):pDUAL-GFH41* |
| B888 | 4C | *h^+^ leu1-32 ura4-D18 ade6-M210 his3-D1 arg3-D4 mis18-262 // leu1:: mis18_MeDiY_:pDUAL-GFH41* |
| B963 | 4C | *h^+^ leu1-32 ura4-D18 ade6-M210 his3-D1 arg3-D4 mis18-262 // leu1:: mis18_MeDiY_(I31A):pDUAL-GFH41* |
| B879 | 4C | *h^+^ leu1-32 ura4-D18 ade6-M210 his3-D1 arg3-D4 mis18-262 // leu1:: mis18_MeDiY_(Y114A):pDUAL-GFH41* |
| B873 | 4D, S4 | *h^+^ leu1-32 ura4-D18 ade6-M210 his3-D1 arg3-D4 // leu1::mis18^+^:pDUAL-GFH41* |
| B874 | 4D, S4 | *h^+^ leu1-32 ura4-D18 ade6-M210 his3-D1 arg3-D4 // leu1::mis18 (I31A):pDUAL-GFH41* |
| B951 | 4D, S4 | *h^+^ leu1-32 ura4-D18 ade6-M210 his3-D1 arg3-D4 // leu1::mis18 (Y114A):pDUAL-GFH41* |
